# Amyloid Beta-Peptide Increases BACE1 Translation through the Phosphorylation of the Eukaryotic Initiation Factor-2*α*

**DOI:** 10.1155/2020/2739459

**Published:** 2020-09-19

**Authors:** Pol Picón-Pagès, Daniela A. Gutiérrez, Alejandro Barranco-Almohalla, Giulia Crepin, Marta Tajes, Gerard ILL-Raga, Francesc X. Guix, Silvia Menéndez, Montserrat Arumí-Uría, Rubén Vicente, Alejandra R. Álvarez, Francisco J. Muñoz

**Affiliations:** ^1^Laboratory of Molecular Physiology, Faculty of Health and Life Sciences, Universitat Pompeu Fabra, Barcelona, Spain; ^2^Cell Signaling Laboratory, Centro UC de Envejecimiento y Regeneración (CARE), Department of Cellular and Molecular Biology, Biological Sciences Faculty, Pontificia Universidad Católica de Chile, Santiago, Chile; ^3^Group of Biomedical Research in Heart Diseases, Hospital del Mar-Medical Research Institute (IMIM), Barcelona, Spain; ^4^Division of Physiological Sciences, Federal University of Espírito Santo, Vitória, Espírito Santo, Brazil; ^5^Department of Molecular Neurobiology, Centro de Biología Molecular “Severo Ochoa”, CSIC-UAM, Madrid, Spain; ^6^Cancer Research Programm, Hospital del Mar-Medical Research Institute (IMIM), Parc de Salut Mar, Barcelona, Spain; ^7^Servicio de Anatomía Patológica, Hospital del Mar, Parc de Salut Mar, Barcelona, Spain

## Abstract

Alzheimer's disease (AD) is tightly linked to oxidative stress since amyloid beta-peptide (A*β*) aggregates generate free radicals. Moreover, the aggregation of A*β* is increased by oxidative stress, and the neurotoxicity induced by the oligomers and fibrils is in part mediated by free radicals. Interestingly, it has been reported that oxidative stress can also induce BACE1 transcription and expression. BACE1 is the key enzyme in the cleavage of the amyloid precursor protein to produce A*β*, and the expression of this enzyme has been previously shown to be enhanced in the brains of Alzheimer's patients. Here, we have found that BACE1 expression is increased in the hippocampi from AD patients at both the early (Braak stage II) and late (Braak stage VI) stages of the disease as studied by immunohistochemistry and western blot. To address the role of A*β* and oxidative stress in the regulation of BACE1 expression, we have analyzed the effect of subtoxic concentrations of A*β* oligomers (0.25 *μ*M) and H_2_O_2_ (10 mM) on a human neuroblastoma cell line. Firstly, our results show that A*β* oligomers and H_2_O_2_ induce an increase of *BACE1* mRNA as we studied by qPCR. Regarding BACE1 translation, it is dependent on the phosphorylation of the eukaryotic initiation factor 2*α* (eIF2*α*), since *BACE1* mRNA bears a 5′UTR that avoids its translation under basal conditions. BACE1 5′UTR contains four upstream initiating codons (uAUGs), and its translation is activated when eIF2*α* is phosphorylated. Consistently, we have obtained that A*β* oligomers and H_2_O_2_ increase the levels of BACE1 and p-eIF2*α* assayed by western blot and confocal microscopy. Our results suggest that A*β* oligomers increase BACE1 translation by phosphorylating eIF2*α* in a process that involves oxidative stress and conforms a pathophysiological loop, where the A*β* once aggregated favors its own production continuously by the increase in BACE1 expression as observed in AD patients.

## 1. Introduction

Alzheimer's disease (AD) is a neurodegenerative process that occurs in the elderly being the most prevalent type of human dementia. AD symptoms consist in dramatic memory deficits and an irreversible cognitive decline. They start with neuronal death in the hippocampus and the brain structure for learning and memory, and later, the neuronal loss progresses to other cortical areas. The histopathological characteristics of AD patients are extracellular senile plaques and intracellular neurofibrillary tangles. The senile plaques are mainly composed of amyloid *β*-protein (A*β*) and the neurofibrillary tangles of hyperphosphorylated tau protein [1, 2].

A*β* is a peptide having from 36 to 43 amino acids [3]; however, A*β*_1-40_ is the most abundant. A*β* is produced by the consecutive enzymatic action of the beta- and gamma-secretase activities on an integral type I transmembrane glycoprotein termed amyloid precursor protein (APP) [4]. A*β*_1-42_ production increases with aging [5], in patients of familiar AD bearing some mutations in the presenilins (PS1/PS2) [6], which are the catalytic core of the gamma-secretase complex, or due to the nitrotyrosination of the gamma-secretase complex [7]. The pathophysiological relevance of this shift in the A*β* species production is due to the high aggregant properties of the A*β*_1-42_ [8, 9], which makes it more neurotoxic.

The oligomeric forms are the responsible for the most neurotoxic effects associated to the A*β* [10–12]. A*β* aggregates produce H_2_O_2_ [13] inducing oxidative stress and starting an intracellular cascade of reactive oxygen species, which will compromise neuronal viability [14, 15]. Moreover, oxidative stress has been reported to increase the transcription and expression of the enzyme Beta-site Amyloid precursor protein Cleaving Enzyme 1 (BACE1) [16, 17]. BACE1 is the key enzyme that carries out the beta-secretase activity on APP to initiate the A*β* production pathway.

BACE1 expression is physiologically repressed at the transcriptional and translational level. There are different pathways, mostly related to different types of stress, that induce the nuclear translocation of transcription factors, such as cJun/cFos, to bind to *BACE1* gene promoter [18, 19]. However, the tightest control of BACE1 is at the translational level since *BACE1* mRNA bears a particular 5′UTR that contains four upstream initiation codons (uAUGs) and possesses a rich GC content. These sequence motifs confer a particular secondary structure to that region of the mRNA that impairs ribosomes to reach the main AUG in order to start BACE1 translation [20–23]. In basal conditions, ribosomes remain in one of the uAUG, especially the second one. *BACE1* mRNA translation is only activated when the eukaryotic initiation factor 2*α* (eIF2*α*) is phosphorylated at serine 51 [19, 24–26]. The physiological relevance of the eIF2*α* phosphorylation is to block the translation of the most of the proteins under stressful conditions and to induce only the translation of a group of special proteins that bear 3 or more uAUGs in the 5′UTR [27]. These stressful conditions are virus infection, reticular stress, nutrient deprivation, or oxidative stress. There are four eIF2*α* kinases: heme-regulated eukaryotic initiation (HRI), general control nonderepressible 2 kinase (GCN2), double-stranded RNA-activated protein kinase (PKR), and double-stranded RNA-activated protein kinase-like (PERK), which phosphorylate eIF2*α* at serine 51 blocking translation initiation [25–30].

## 2. Materials and Methods

### 2.1. BACE1 Expression Study by Immunohistochemistry in Human Hippocampi

Human hippocampal samples were supplied by the Neurological Tissue Bank of the Biobank-Hospital Clínic-IDIBAPS, Barcelona, Spain. The procedure was carried out according to the rules of the Helsinki Declaration and to the Ethics Committee of the Institut Municipal d'Investigacions Mèdiques-Universitat Pompeu Fabra (EC-IMIM-UPF). Hippocampal samples were obtained from 3 nondemented controls (1 man and 2 women; mean: 69 years old), 2 AD patients at Braak stage II (2 men; 83 and 76 years old), and 4 AD patients at Braak stage VI (2 men and 2 women; mean: 70 years old). Sections (5 *μ*m) were treated with 4% H_2_O_2_ and incubated o.n. at 4°C with 1 : 100 rabbit anti-BACE1 Antibody (Ab; Invitrogen). The secondary Ab was 1 : 500 donkey anti-rabbit peroxidase-conjugated Ab, which was incubated for 1 h at room temperature (RT). A Peroxidase Substrate Kit DAB (Vector) was used. Slides were stained with hematoxylin and fixed. The images were taken by a Leica DMR microscope.

### 2.2. BACE1 Expression Study by Western Blot in Human Hippocampi

Human brain tissue sections, obtained as indicated in the subsection 2.1., were lysed with 50 *μ*L RIPA buffer: 150 mM sodium chloride, 1% Triton X-100 0.5% sodium deoxycholate, 0.1% sodium dodecyl sulfate (SDS), 50 mM Tris-HCl, 1 mM dithiothreitol, 1 mM sodium orthovanadate, and protease inhibitor cocktail (Roche), pH 8. Membranes were blocked for 1 h at RT with Tween 20-Tris buffer solution (TTBS; 100 mM Tris-HCl, 150 mM NaCl, pH 7.5) plus 5% skimmed milk. Then, membranes were incubated overnight (o.n.) at 4°C with 1 : 4,000 mouse anti-actin Ab (Sigma) or 1 : 1,000 rabbit anti-BACE1 Ab. 1 : 2,000 secondary Abs were horseradish peroxidase-conjugated donkey anti-mouse and anti-rabbit (GE Healthcare), which were incubated for 1 h at RT. Bands were visualized with Super Signal (Pierce) and analyzed with the Quantity One system in a BioRad Universal Hood II.

### 2.3. Cell Line

Human neuroblastoma cells (SH-SY5Y cells) were grown with Ham's F12 GlutaMax (F12 medium; Gibco) supplemented with 15% fetal bovine serum (FBS; Gibco) and 1% penicillin/streptomycin (Gibco). Cells were incubated at 37°C in a humidified atmosphere of 5% CO_2_.

### 2.4. A*β* Oligomer Formation

1 mg of lyophilized human A*β*_1-42_ (Anaspec) went to solution with 250 *μ*L of ultrapure MilliQ water. Therefore, we adjusted pH to ≥10.5 with NaOH (1 M) to keep away from the A*β* isoelectric point. The solution was sonicated for 1 min in 250 *μ*L of phosphate buffer (20 mM; pH 7.4). To favor the proper oligomer formation, the A*β* aliquots were dissolved in F12 medium at 0.4 mg/mL and incubated for 24 at 4°C.

### 2.5. Cell Viability Studies

SH-SY5Y cells were seeded in a 96-well plate at a density of 2.5 × 10^4^ cells/well. After 12 h, the growth medium was removed. Cells were treated with increasing concentrations of A*β*_1-42_ oligomers or H_2_O_2_ (Sigma**)** in F12 medium. Treatments were carried out along 24 h at 37°C. Then, cell survival was assayed by 3-(4,5-dimethylthiazol-2-yl)-2,5-diphenyltetrazolium bromide (MTT) reduction method. It consists of the addition of 10% (regarding cell medium volume) of MTT stock solution at 5 mg/mL. Cells were incubated with the MTT for 2 h. Medium was discarded and 100 *μ*L of DMSO were placed per well. MTT absorbance was measured in an absorbance plate reader (BioRad) at A540 nm and A650 nm (as reference). Control cells treated with phosphate buffer saline (PBS) were the 100%. Bridge field images were obtained with a Leica DM IL microscope.

### 2.6. Apoptosis Studies

Cells were seeded on coverslips in 24-well plates at a density of 3 × 10^4^ cells/well. After 12 h, the growth medium was removed and cells were treated for 24 h with subtoxic concentrations of A*β*_1-42_ oligomers (0.25 *μ*M) or H_2_O_2_ (10 *μ*M) and the toxic ones for A*β*_1-42_ oligomers (15 *μ*M) and H_2_O_2_ (100 *μ*M) in F12 medium. Cells were fixed with 4% paraformaldehyde (PFA) and permeabilized with 0.1% Triton X-100 at RT. Coverslips were incubated o.n. at 4°C with 1 : 100 rabbit anti-cleaved Caspase-3 Ab (Cell Signalling). Cells were washed thrice and incubated with 1 : 2,000 Alexa Fluor 555 goat anti-rabbit Ab (Life Technologies) and Hoechst 1 : 10,000 (Thermo Scientific) for 1 h at RT. Coverslips were mounted with Fluoromount (Southern Biotech). Digital images were taken with a Leica TCS SP confocal microscope and analyzed with the Leica confocal software.

### 2.7. Transcriptional Studies in Cells

Cells were seeded on 60 mm Petri dishes at a density of 6 × 10^5^ cells/dish. After 12 h, the growth medium was removed and cells were treated for 24 h with subtoxic concentrations of 0.25 *μ*M A*β*_1-42_ oligomers or 10 *μ*M H_2_O_2_ in F12 medium. The mRNA was extracted by using the NucleoSpin RNA extraction kit (Macherey Nagel) and quantified with NanoDrop ND-1000 (Thermo Fisher Scientific). *BACE1* and *HPRT* cDNAs were obtained with the SuperScript III Reverse Transcriptase (Invitrogen). Finally, a quantitative PCR was performed by using the fluorophore Sybr Green (Thermo Fisher Scientific). Samples were quantified with QuantStudio 12K Flex Real-Time PCR System (Thermo Fisher Scientific).

### 2.8. Translational Studies by Western Blot in Cells

Cells were seeded on 6-well plates at a density of 3 × 10^5^ cells/well. After 12 h, the growth medium was removed and cells were treated for 24 h with subtoxic concentrations of 0.25 *μ*M A*β*_1-42_ oligomers or 10 *μ*M H_2_O_2_ in F12 medium. To study phosphorylated proteins, the cells were lysed on ice with lysis solution as indicated in subsection 2.2. Extracts were homogenised using vortex for 30 min at 4°C; afterwards, samples were centrifuged at 10,000 for 5 min to obtain the supernatant. A Bio-Rad kit was used to calculate protein concentrations. Aliquots of 20 *μ*L (for phosphorylated proteins) or 80 *μ*g (for the other proteins) were loaded into a 10% SDS-PAGE gels. Afterwards, proteins were transferred onto 0.2 *μ*m pore nitrocellulose membranes. Membranes were blocked for 1 h at RT with TTBS plus 5% bovine serum albumin (BSA) for phosphorylated proteins or 5% skimmed milk for the other proteins. Then, membranes were incubated o.n. at 4°C with 1 : 1,000 rabbit anti-BACE1 Ab (Invitrogen), 1 : 500 rabbit anti-p-eIF2*α* (Ser51) Ab (Invitrogen), 1 : 500 mouse anti-eIF2*α* Ab (Abcam), and 1 : 5,000 mouse anti-tubulin Ab (Sigma). 1 : 2,000 secondary Abs were horseradish peroxidase-conjugated donkey anti-mouse and anti-rabbit (GE Healthcare) for 1 h at RT. Bands were visualized with Super Signal (Pierce) and analyzed with the Quantity One system in a BioRad Universal Hood II.

### 2.9. Translational Studies by Confocal Microscopy in Cells

Cells were seeded on coverslips in 24-well plates at a density of 3 × 10^4^ cells/well. After 12 h, the growth medium was removed, and cells were treated for 24 h with subtoxic concentrations of 0.25 *μ*M A*β*_1-42_ oligomers or 10 *μ*M H_2_O_2_ in F12 medium plus 15% FBS. Then, cells were fixed with 4% paraformaldehyde (PFA). Cells were permeabilized with 0.1% Triton X-100. Coverslips were incubated o.n. at 4°C with 1 : 100 mouse anti-eIF2*α* Ab, rabbit anti-p-eIF2*α* Ab, or rabbit anti-BACE1 Ab. After primary Abs, cells were incubated with 1 : 2,000 Alexa Fluor 555 goat anti-rabbit Ab or 1 : 2,000 Alexa Fluor 647 goat anti-mouse Ab (Life Technologies) for 1 h at RT. Coverslips were mounted with Fluoromount. Digital images were taken with a Leica TCS SP confocal microscope and analyzed with Leica confocal software. Immunofluorescence was quantified by ImageJ program.

### 2.10. Statistical Analysis

Data are expressed as mean ± SEM of n experiments as indicated in the corresponding figures. Statistical analyses were performed by one-way ANOVA using Tukey's posttest or Student's **t**-test using the GraphPad software.

## 3. Results

### 3.1. BACE1 Expression Is Increased in the Hippocampi from AD Patients

**Figure 1:**
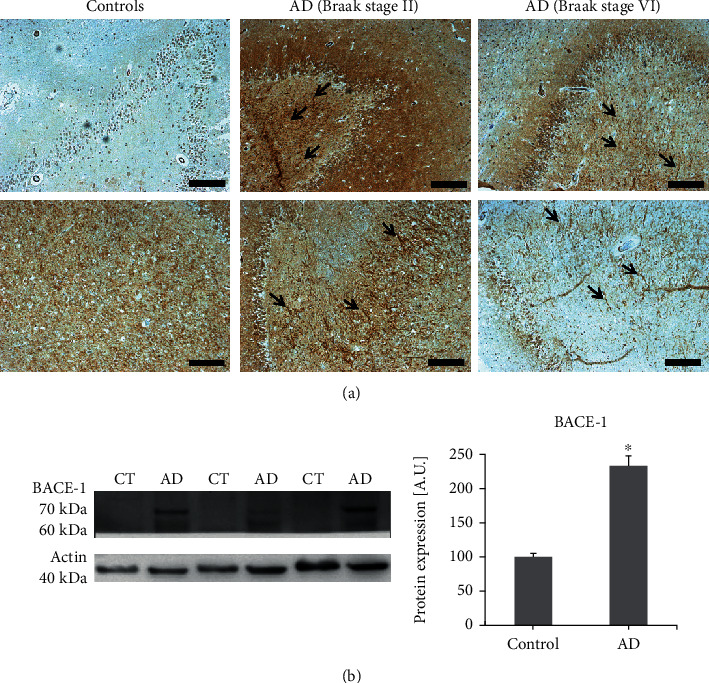
BACE1 expression increases in the hippocampi from AD patients. (a) The expression of BACE1 was studied by immunohistochemical analysis in hippocampal sections from nondemented controls and AD patients at the initial stages of the disease (Braak stage II) and late stages of the disease (Braak stage VI). Here, we show the representative images obtained in two different individuals from each group. The expression of BACE-1 (brown) was predominant along the axons from AD patients as indicated by black arrows. Bars represent 200 *μ*m. (b) BACE1 expression was also studied by western blot from hippocampal samples of nondemented controls and AD patients. Data are expressed as A.U. and represent mean ± SEM of 4 samples per each group. ^∗^*p* < 0.05*vs.* nondemented controls by Student's *t*-test. BACE1 bands correspond to the mature glycosylated enzyme (70 kDa) and the immature enzyme (~60 kDa).

There are previous works that reported increased expression of BACE1 in AD patients [31–33] relating this finding with the enhanced A*β* production that will induce the onset and progression of the disease. Consistently, A*β*_1-42_ oligomers have been reported to be present in the hippocampi from AD patients since the early stages of the disease [34]. Here, we have studied the expression of BACE1 in the hippocampi from AD patients at two stages of the disease: Braak II (one of the initial stages showing the early symptoms of AD) and Braak VI (the final stage of the disease, when the A*β* is widespread in the brain and dementia is severe). We found in both stages an increased expression of BACE1 (Figure 1(a)), linking the early enhancement of BACE1 expression with A*β* production. Furthermore, this increment in BACE1 expression was also demonstrated through western blot (Figure 1(b)) when the mature glycosylated form (70 kDa) was analysed. AD patient samples showed an increased expression of BACE1 compared with nondemented controls (*p* < 0.05). The maintained BACE1 expression even in the late stage of the disease suggest that oxidative stress, which has been reported to be significantly increased in AD [14], would be inducing BACE1 expression directly or by the indirect effect of A*β*_1-42_ oligomers, which generate free radicals, as we address in the present work.

### 3.2. A*β*_1-42_ Oligomers and H_2_O_2_ Decrease Cell Viability at High Concentrations

**Figure 2:**
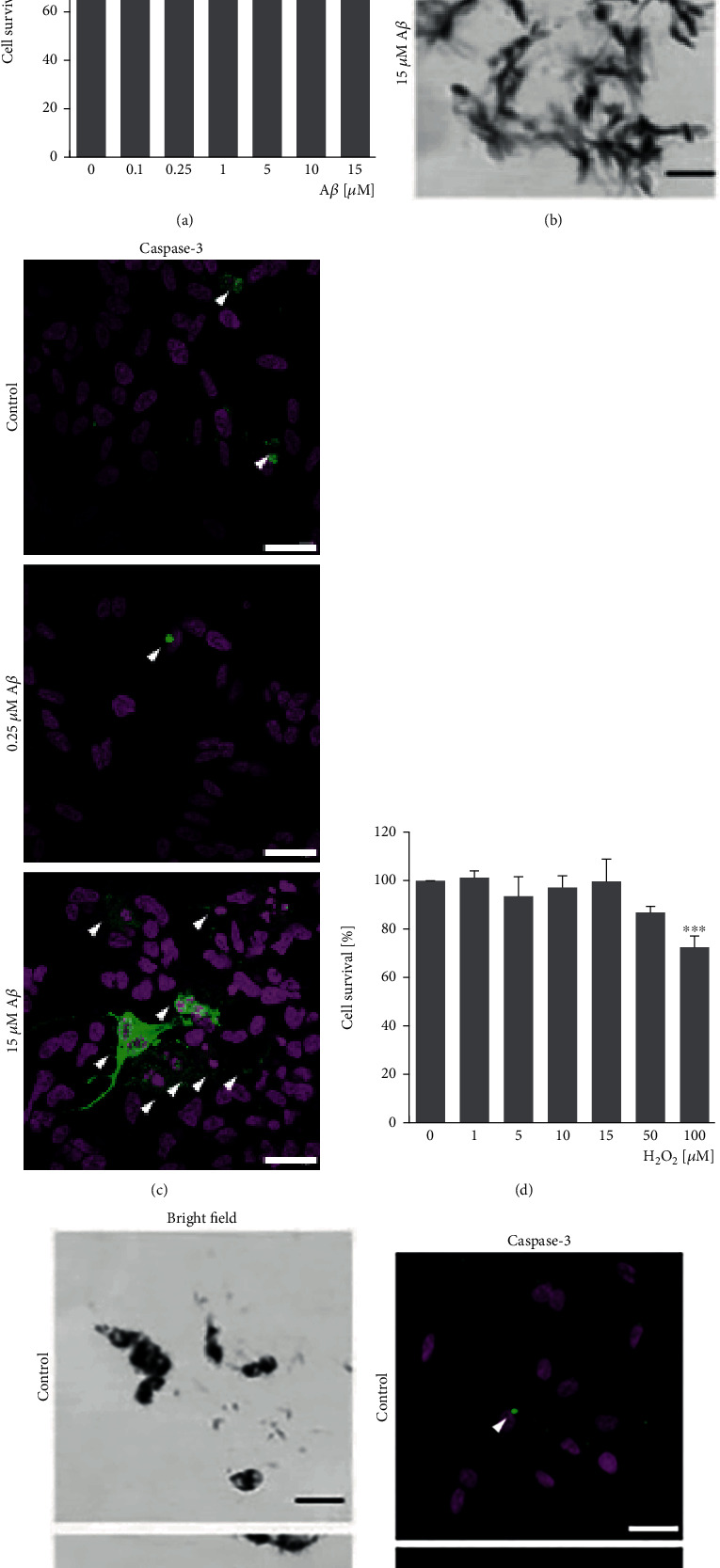
A*β*_1-42_ oligomers and H_2_O_2_ decrease cell viability. Human neuroblastoma cells were treated with increasing concentrations of (a) A*β*_1-42_ oligomers or (d) H_2_O_2_ for 24 h. Cell survival was measured by MTT reduction. Data are mean ± SEM of 4 independent experiments performed in triplicate. ^∗∗^*p* < 0.005, ^∗∗∗^*p* < 0.001*vs.* control by one-way ANOVA using Tukey's posttest. Images of cells with the MTT reduced to blue formazan were taken after 24 h of treatment with (b) A*β*_1-42_ oligomers or (e) H_2_O_2_. Bars represent 20 *μ*m. The proapoptotic state of the cells was studied by the identification of cleavaged caspase-3 (green; marked with white arrows) after 24 h of treatment with (c) A*β*_1-42_ oligomers or (f) H_2_O_2_. Nuclei were counterstained with Hoechst (pink). Bars represent 30 *μ*m.

Human neuroblastoma cells were treated with increased concentration of oligomeric A*β*_1-42_ for 24 h (Figure 2(a)) in order to study their neurotoxic abilities and to find the range of concentrations that do not produce a significant cytotoxicity to carry out the experiments on BACE1 regulation. A*β*_1-42_ oligomers were significantly neurotoxic at 5 *μ*M (*p* < 0.005), 10 *μ*M (*p* < 0.001), and 15 *μ*M (*p* < 0.001). The production of H_2_O_2_ by the A*β*_1-42_ oligomers is a continuous process, while the A*β*_1-42_ is present, along 24 h in our experiments. Therefore, we have also studied the effect of the treatment with H_2_O_2_ on cell viability (Figure 2(d)). We have obtained that H_2_O_2_ is cytotoxic at 100 *μ*M (*p* < 0.001).

The cell cytotoxicity was also studied by images obtained after the reduction of the MTT to blue formazan by bright field microscopy (Figures 2(b) and 2(e)) and with an Ab against active caspase-3 (Figures 2(c) and 2(f)). In both studies, 15 *μ*M A*β*_1-42_ oligomers and 100 *μ*M H_2_O_2_ showed a low reduction of MTT and high caspase activation as expected.

In order to mimic the pathophysiological effects of a continuous and insidious damage on neurons along the life, we decided to carry out the experiments on BACE1 regulation with the subtoxic concentration of 0.25 *μ*M A*β*_1-42_ oligomers and 10 *μ*M H_2_O_2_. These concentrations were producing neither cytotoxicity when assayed by MTT reduction (Figures 2(a), 2(d), 2(b), and 2(e)) nor significant caspase activation (Figures 2(c) and 2(f)).

### 3.3. A*β*_1-42_ Oligomers and H_2_O_2_ Increase BACE1 Transcription and Translation

**Figure 3:**
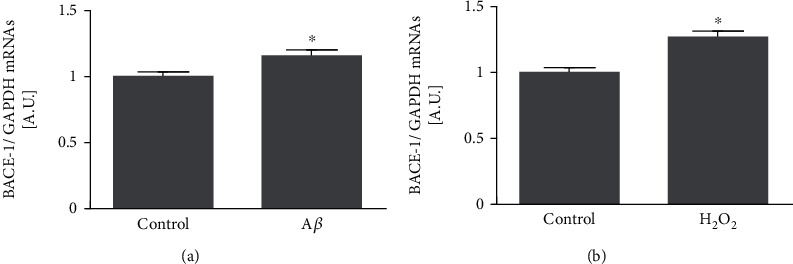
A*β*_1-42_ oligomers and H_2_O_2_ increase BACE1 transcription. Human neuroblastoma cells were treated with subtoxic concentrations of A*β*_1-42_ oligomers (0.25 *μ*M) or H_2_O_2_ (10 *μ*M) for 24 h. *BACE1* mRNAs were quantified by qPCR after (a) A*β*_1-42_ oligomers or (b) H_2_O_2_ treatments. Data are expressed as arbitrary units (A.U.) and represent mean ± SEM of 3 independent experiments performed by triplicate. ^∗^*p* < 0.05*vs.* control cells by Student's *t*-test.

Human neuroblastoma cells were treated with 0.25 *μ*M A*β*_1-42_ oligomers for 24 h, and *BACE1* mRNA showed a significant transcriptional increase (Figure 3(a); *p* < 0.05). The treatment with 10 *μ*M H_2_O_2_ for 24 h produce the same effect on *BACE1* mRNA transcription (Figure 3(b); *p* < 0.05). These results suggest that both A*β*_1-42_ oligomers and H_2_O_2_ share common mechanisms at the transcriptional level.

**Figure 4:**
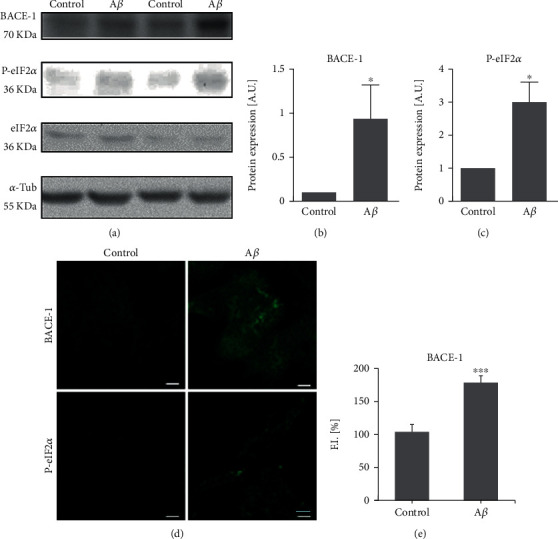
Subtoxic A*β*_1-42_ oligomers increase BACE1 translation. Neuroblastoma cells were treated with subtoxic concentrations of A*β*_1-42_ oligomers (0.25 *μ*M) for 24 h. BACE1, p-eIF2*α*, and eIF2*α* expressions were studied by (a) western blot and (d) immunofluorescence. The bands corresponding to (b) BACE1/tubulin and (c) p-eIF2*α*/eIF2*α* were quantified and expressed as A.U. Data are expressed as arbitrary units (A.U.) and represent mean ± SEM of 4-7 independent experiments. ^∗^*p* < 0.05*vs.* control cells by Student's *t*-test. BACE1 expression by immunofluorescence analysis was quantified (e) and expressed as fluorescence intensity (F.I.). Data are mean ± SEM of 3 independent experiments. ^∗∗∗^*p* < 0.001*vs.* control cells by Student's *t*-test. Bars represent 5 *μ*m.

**Figure 5:**
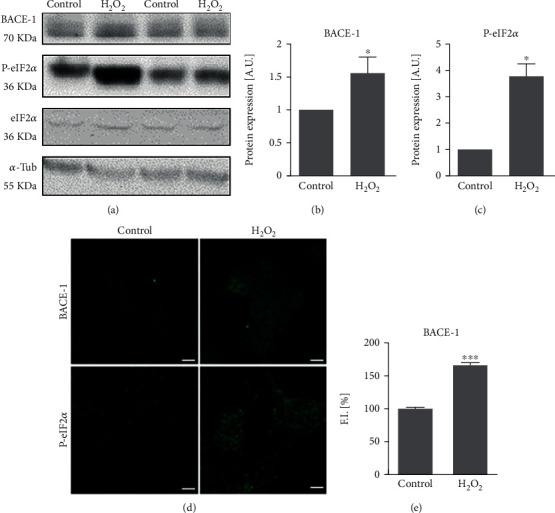
Subtoxic H_2_O_2_ increases BACE1 translation. Human neuroblastoma cells were challenged with subtoxic concentrations of H_2_O_2_ (10 *μ*M) for 24 h. BACE1, p-eIF2*α*, and eIF2*α* expressions were studied by (a) western blot and (d) immunofluorescence. The bands corresponding to (b) BACE1/tubulin and (c) p-eIF2*α*/eIF2*α* were quantified and expressed as arbitrary units (A.U.). Data are mean ± SEM of 3-9 independent experiments. ^∗^*p* < 0.05*vs.* control cells by Student's *t*-test. BACE1 expression by immunofluorescence analysis was quantified (e) and expressed as fluorescence intensity (F.I.). Data are mean ± SEM of 3 independent experiments. ^∗∗∗^*p* < 0.001*vs.* control cells by Student *t*-test. Bars represent 5 *μ*m.

Attending to the effects on BACE1 translation, we have obtained that 0.25 *μ*M A*β*_1-42_ oligomers increased BACE1 expression after 24 h as it was analyzed by western blot (Figure 4; *p* < 0.05 by western blot and *p* < 0.001 by immunofluorescence). Similar results were obtained when cells were treated with 10 *μ*M H_2_O_2_ for 24 h (Figure 5; *p* < 0.05 by western blot and *p* < 0.001 by immunofluorescence). Interestingly, the phosphorylation of eIF2*α* was increased with both 0.25 *μ*M A*β*_1-42_ oligomers and 10 *μ*M H_2_O_2_ (Figures 4, 5; *p* < 0.05 by western blot) supporting that BACE1 expression is due to an increase in its translation. These results also correlated with the increased fluorescence showed by p-eIF2*α* after the treatment with 0.25 *μ*M A*β*_1-42_ oligomers and 10 *μ*M H_2_O_2_ (Figures 4(d) and 5(d)).

## 4. Discussion

There are many evidences that relate oxidative stress with the ethiopathogenesis of AD, a devastating neurodegenerative disease whose major risk factor is aging. This tight relationship starts with the aggregation of A*β*, which increases by oxidative stress [35]. Once the fibrils are formed, they also produce H_2_O_2_ [13] and hydroxyl radical [36, 37]. In fact, the neurotoxicity of A*β* aggregates has been reported to be mediated by oxidative stress [14, 15, 38, 39]. Moreover, oxidative stress has been demonstrated to increase BACE1 transcription and translation [16, 17]. We have demonstrated in this work that BACE1 expression in increased even in the early stages of the disease. Since oxidative stress is a concomitant process with aging and A*β* aggregation is considered the key factor of AD, according to the amyloid cascade hypothesis, we have studied the effect of A*β* oligomers and oxidative stress in BACE1 expression and the mechanisms that control its pathophysiological expression.

BACE1 is an enzyme that accomplishes some physiological functions as dendritic spine growth in the hippocampus where contributes to memory formation [26]. In fact, BACE1 knockout mice show synaptic plasticity deficits and cognitive impairment [40, 41]. However, the dysregulation of BACE1 has harmful effects. In our study, we have found that long treatments with low nanomolar concentration of A*β*_1-42_ oligomers, mimicking the environment of neurons in AD patients, increase BACE1 transcription and expression in vitro. The relevance of this finding is supported by the increased expression of BACE1 found in AD hippocampi. This would suggest the existence of a loop of amyloid production that will activate BACE1 to release more amyloid contributing to accelerate the dysregulation of BACE1 and the characteristic amyloidosis of AD. This effect should be based in the capability of A*β*_1-42_ oligomers to produce oxidative stress since similar results has been obtained when we used hydrogen peroxide.

BACE1 expression is controlled physiologically by the phosphorylation of eIF2*α* carried out by the enzyme HRI [26]. HRI is activated throughout the nitric oxide released after NMDA receptors activation in glutamatergic neurons. However, aging produces an increase in oxidative stress in brain [42, 43] and would result in the loss of BACE1 physiological control. It is due to the fact that HRI, PKR, and PERK, three of the four kinases that phosphorylates eIF2*α*, can be activated by oxidative stress [44–48]. In our study, we have found that both oxidative stress and A*β*_1-42_ oligomers yield to the phosphorylation of the eIF2*α* in vitro. The dysregulation of the different eIF2*α* kinases by oxidative stress would explain these results.

Summarizing, our results suggest that oxidative stress induced by A*β* oligomers increase BACE1 transcription and translation. Our data support that the mechanism involved in the increased BACE1 expression is the dysregulation of the phosphorylation of the eIF2*α* that would generate the amyloid burden in AD.

## Data Availability

All the data used to support the findings of this study are available from the corresponding author upon request.

## Abbreviations

Ab: Antibody
A*β*: Amyloid-*β* peptide
AD: Alzheimer's disease
APP: Amyloid precursor protein
A.U: Arbitrary units
BACE1: *β*-site APP Cleaving Enzyme 1
BSA: Bovine serum albumin
EIF2*α*: Eukaryotic initiation factor 2-*α*
FBS: Fetal bovine serum
GCN2: General control nonderepressible-2 kinase
HRI: Heme-regulated eukaryotic initiation factor eIF2 *α* kinase
MTT: 3-(4,5-dimethylthiazol-2-yl)-2,5-diphenyltetrazolium bromide
PBS: Phosphate buffer saline
o.n: Overnight
PERK: PKR-like endoplasmic reticulum-related kinase
PKR: Double-stranded RNA-activated protein kinase
PS: Presenilin
RT: Room temperature
SDS: Sodium dodecyl sulphate
TTBS: Tween 20-Tris buffer solution
5′UTR: 5′untranslated region
